# Lichen planus of the external auditory canal: Treatment options and review of literature

**DOI:** 10.1002/ccr3.3049

**Published:** 2020-06-18

**Authors:** Francesco Lazzerini, Luca Bruschini, Stefano Berrettini, Andrea De Vito, Francesca Forli

**Affiliations:** ^1^ ENT Audiology and Phoniatric Unit University Hospital of Pisa Pisa Italy; ^2^ Division of ENT Diseases Karolinska Institute Stockholm Sweden

**Keywords:** bone‐anchored hearing device, dermatology, ear, external auditory canal stenosis, lichen planus, nose and throat

## Abstract

Medical topic therapies can relieve symptoms associated with lichen planus of external auditory canal. Further, bone‐anchored hearing devices represent an optimal solution for hearing restoration in otic lichen planus.

## Graphical abstract

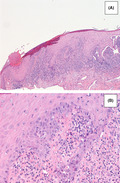

## 1 INTRODUCTION

Lichen planus (LP) is a common inflammatory chronic disease of the skin and mucosae.^1^ It is caused by a T cell–mediated immune response directed toward keratinocytes expressed on their surface heterogeneous antigens of different nature: toxic (eg, from drugs), viral (eg, HBV and HCV), or haptenic. Usually, LP involves not only the skin, typically on flexural surfaces of the limbs, but also the mucosae are frequently affected, in the oral and genital district.^2^

Even if 0.3%‐0.8% of the population is reported to present some forms of lichen planus, the otic localization of LP is thought to be extremely rare and only two single case reports^3^, ^4^ and two small case series^5^, ^6^ are published in the scientific literature, for a total of 24 reported patients.

The specific management strategy for this rare disease is unclear, and medical and surgical therapies have been proposed. Otic LP, indeed, presents two main problems: the stenosis of the external auditory canal and an associated conductive hearing loss.^4^, ^6^

We report a case of oticus LP with mixed hearing loss; the clinical presentation, diagnostic issues, and treatment are discussed. Moreover, we propose a new approach for the treatment of this disease.

## 2 CASE HISTORY/ EXAMINATION

**Figure 1:**
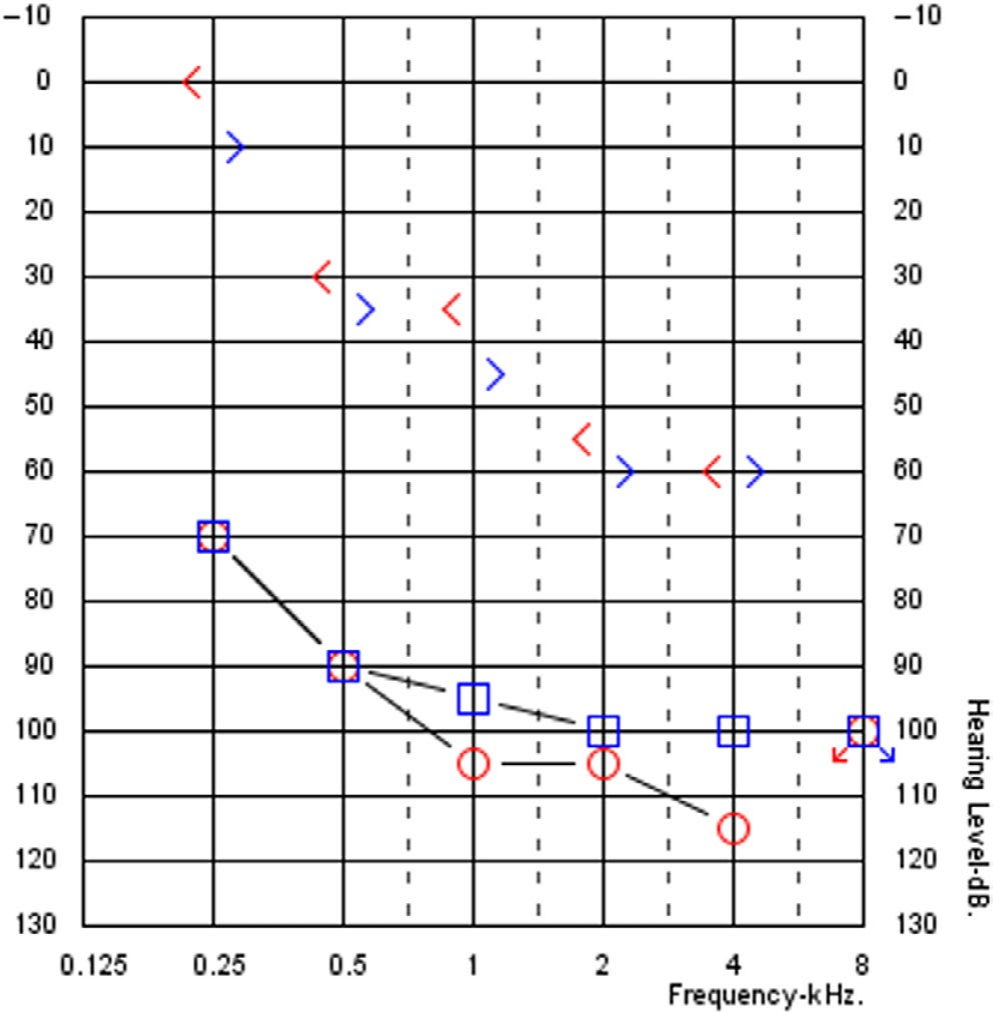
Pure tone audiometry of the patient that shows a bilateral mixed hearing loss

**Figure 2:**
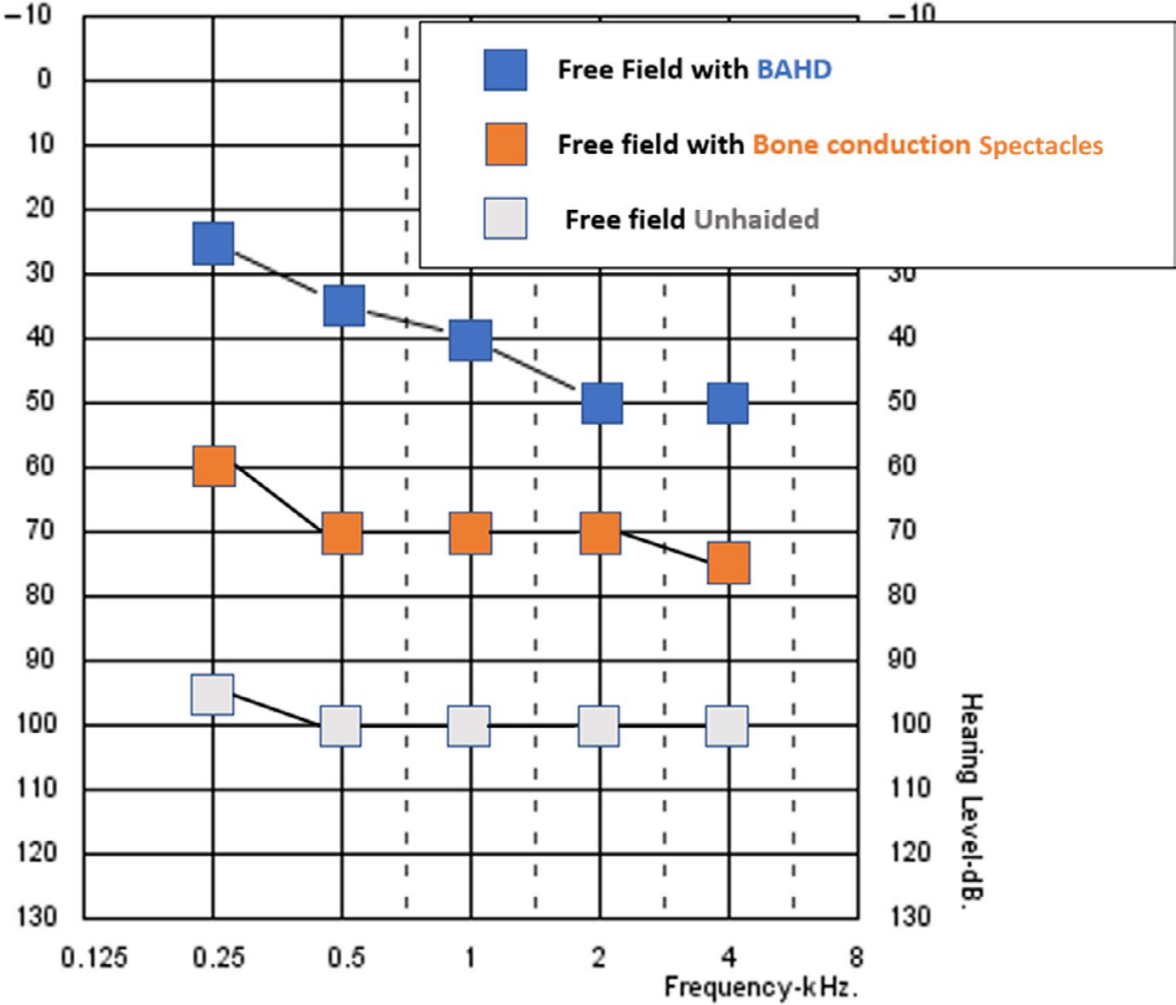
Free field audiometry of the patient, aided and unaided

PM, a 78‐year‐old woman, was admitted to our audiologic service for bilateral ear discharge, ear pruritus, and pain and progressive hearing loss, developed 5 years before, but rapidly worsened in the last 12 months. In the last 6 months, the patient had been using bone conduction hearing aid spectacles with very limited benefit. The otomicroscopy and the otoendoscopy showed an easy to blood, totally stenotic external auditory canal (EAC) bilaterally. Tympanic membrane was not visible, bilaterally. Tonal audiometry revealed a severe‐to‐profound mixed bilateral hearing loss, with an air conduction pure tone average 500‐1000‐2000‐4000 Hz (PTA) of 96.25 dB on the left side and 103.75 dB on the right side and a bone conduction PTA of 50 dB on the left side and 45 dB on the right side (Figure 1). The free field unaided audiometry showed a PTA of 100 dB and a PTA of 70 dB with the bone conduction hearing aid spectacles (Figure 2). The open‐set disyllabic word recognition score with bone conduction hearing aid spectacles was 30% in silence and 15% with background noise (SNR + 10); further, the patient showed no open‐set speech recognition abilities unaided.

### 2.1 Differential diagnosis, investigations, and treatment

**Figure 3:**
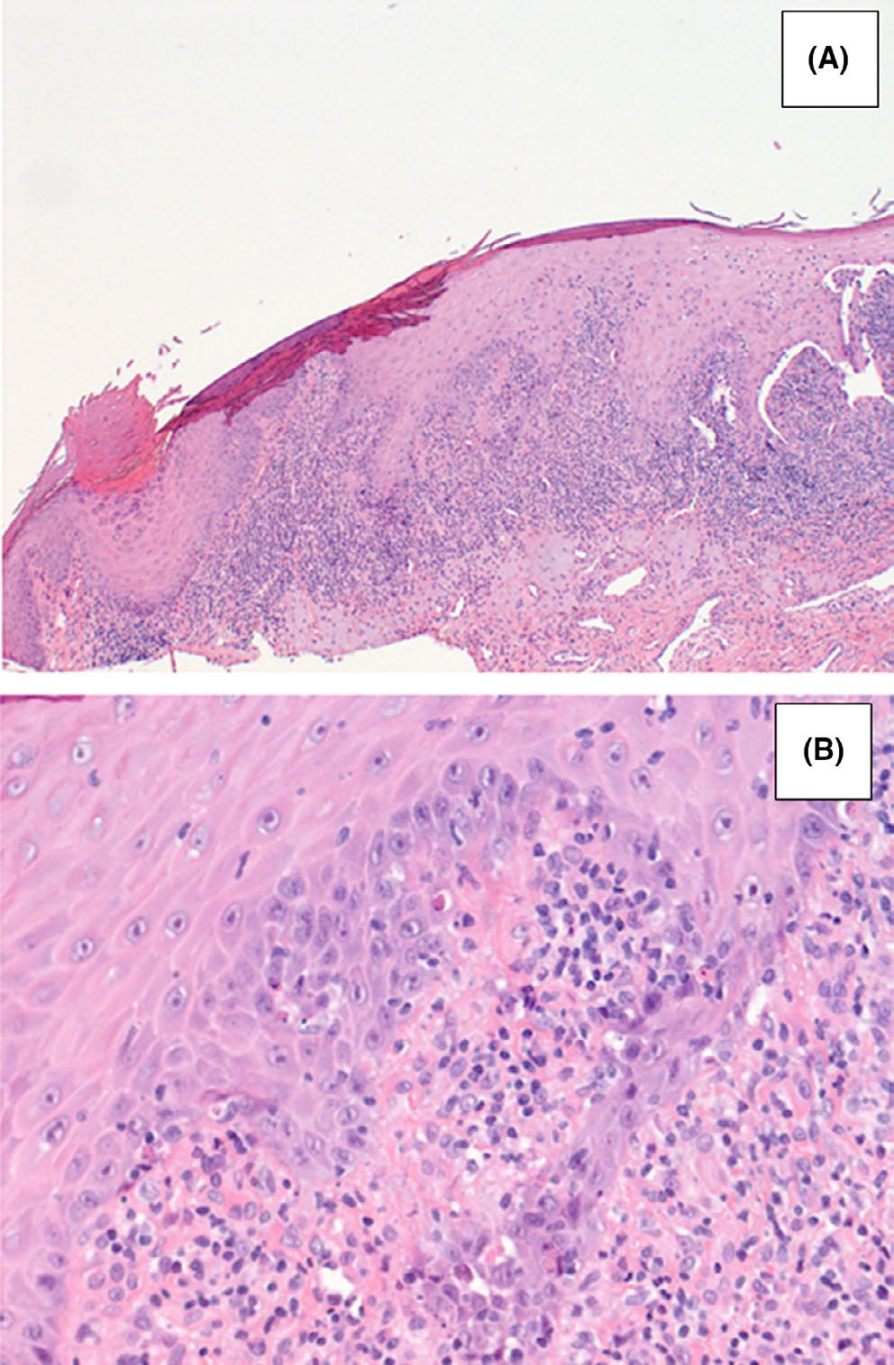
Histopathologic examination of EAC biotic sample after hematoxylin and eosin staining, showing hyperkeratosis, hypergranulosis, irregular acanthosis, apoptosis of keratinocytes, lymphohistiocytic infiltration (A ×100, B ×400)

**Figure 4:**
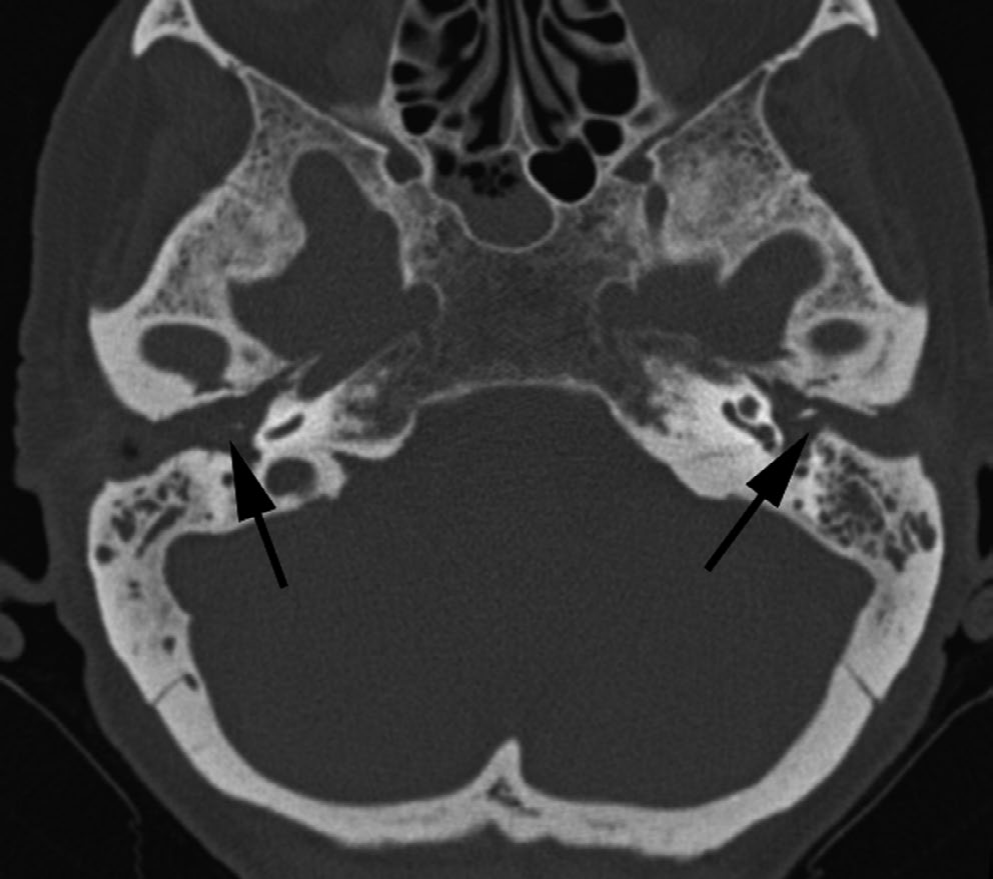
Axial computed tomography (CT) scans of the middle ear. Black arrows showing a bilateral EAC stenosis and isodense tissue occupying the whole middle ear, bilaterally

The anamnesis revealed that the patient was affected by oral lichen planus, developed after a mourning occurred 12 months before, and no other relevant comorbidities. In the suspect of otic LP, we performed a biopsy of the EAC tissue, under local anesthesia. The histopathologic examination confirmed the diagnosis of lichen planus of the EAC (Figure 3). Furthermore, we submitted the patient to a computed tomography (CT) of the temporal bone. The imaging revealed an important amount of isodense soft tissue occupying bilaterally the EAC and the whole middle ear (Figure 4).

In order to treat the otic dermatologic manifestations, we prescribed an otologic topic therapy with ciprofloxacin‐dexamethasone drops (3 mg/1 mg/ml 5 drops in both ears, once a day for 3 months).

Further, in order to improve hearing results, we proposed a bone‐anchored hearing device (BAHD) implantation surgery. We did not propose a cochlear implant procedure for the age of the patient and for the middle ear involvement by the inflammatory disease. After a complete preoperatory counseling and a test with the bone conduction softband device, the patient gave her informed consent to the procedure. The left side was chosen because of patient subjective preference. During the device test on softband, indeed, no significant objective differences were noticeable between left and right sides at free field audiometry or open‐set perception evaluation. We implanted the device abutment, and after one month, we activated and fitted the device.

Considering the patient's bone conductive hearing threshold, we proposed the most powerful hearing processor that was available on the market, indicated for a bone conductive PTA better than 65 dB.

### 2.2 Outcome and follow‐up

After 3 months from the activation of the BAHD and two fitting sessions, the patient had a PTA of about 40 dB HL at free field audiometry and an open‐set disyllabic word recognition score of 70% in silence and 35% with background noise with device on (see Figure 2). Further, after 2 weeks of treatment with the otologic eardrops, the patient reported a drastic reduction in the ear discharge bilaterally. No more pruritus or ear pain has been ever reported by the patient. During the otoscopic follow‐up with otomicroscopy, we noticed a progressive decrease in the inflammation of EAC tissues; nevertheless, at the bottom of EAC, a thick layer of tissues replacing the normal tympanic membrane always remained noticeable.

Nowadays, the patient continues with the local therapy cyclically for 20 days each month. As at the time of writing this paper, the follow‐up duration was 12 months.

## 3 DISCUSSION

Otic LP is an extremely rare localization of LP, with few cases reported in the literature. Clinically, it is characterized by a conductive hearing loss, running ear, pruritus, pain, bleeding from the EAC, and tinnitus. The otologic involvement is reported to be bilateral in 14 cases (58,3%) and monolateral in 10 cases (41,6%).

The diagnosis is clinical, with a coexistence of mucosal LP and conductive hearing loss associated with EAC stenosis.^3^, ^4^, ^5^, ^6^ A bioptic sampling of the EAC can confirm the diagnosis of lichen, showing hyperacanthosis, hypergranulosis, dermal lymphocytic infiltrate, focal exocytosis, and damaged basal cell layer with colloid bodies.^3^

In the few cases reported in the scientific literature, medical and surgical therapies have been proposed for the otologic management. Martin et al in one case proposed a surgical treatment with the removal of the inflammatory tissue and the calibration of the external auditory canal and, after the three months of relapsing, a medical therapy with oral acitretin (initially 25 mg/d and then 35 mg/d) followed by oral prednisolone (1mg/Kg/day) with a temporary clinical improvement of the otologic finding and the conductive hearing loss.^3^ Hopsu and Pitkäranta reported three mild cases treated with otologic eardrops with antiseptic and/or corticosteroids resulting in a nonprogression of the otologic finding and a stabilization of hearing threshold.^5^ In a review, Sartori‐Valinotti et al reported seventeen patients undergone a topical otologic therapy with tacrolimus and two patients that received topical clobetasol propionate or a combination of otologic ciprofloxacin and dexamethasone drops with a good rate of subjective and/or objective improvement.^6^ Systemic therapy has been reserved for the patients with severe extra‐otic LP; in this case series are also reported some patients that were previously unsuccessfully submitted to meatoplastic or tympanoplasty surgery, before the diagnosis of LP.^6^ In a recent article, Kosec et al reported one case of unilateral otic LP treated with a meatoplasty in general anesthesia and, because a five years later recurrence, with a canal wall–down tympanoplasty after the fails of the medical treatments with topical and oral steroids.^4^ Globally, the surgical treatment is reported to have controversial results, usually with short‐term benefit.^6^

The patient we report was treated with otologic drop achieving a good control of the local symptoms after twelve months of medical therapy.

Regarding the hearing deficit associated with otic LP, a conductive or mixed hearing loss, with a variable degree of presentation, but frequently mild, is generally reported, due to the stenosis of the EAC and in some cases of the tympanic cavity. Sartori‐Valinotti et al reported the hearing loss to as the most frequent symptom in patients with otic LP, both conductive and mixed; they also report that four patients presenting a relevant hearing loss in their cohort received bilateral hearing aids.^6^ Also, Kosec et al report a case of otic LP with mixed hearing loss, but no strategies for hearing remediation are ever been reported.^4^ Moreover, in the available literature some surgical approaches to settle the stenosis of the ear canal are reported, but results in terms of hearing restorations are limited.^3^, ^4^, ^5^, ^6^

As we previously stated, some authors reported that patients may have benefit from the use of traditional hearing aids^6^; anyway, in some cases, as in the patient herein reported, the EAC is severely stenotic or occluded and an earmold cannot be fitted. Furthermore, it has to be considered that, even with an adequate ventilation, a stenotic and inflammatory EAC can be difficult to receive the earmold. Finally, the chronic trauma by the earmold could precipitate a recurrence of the otologic symptoms in patients that presented a remission of disease. For these reasons, we believe that the implantation of a BAHD is an option to be considered in these cases, and even if nowadays the BAHDs are commonly used for the treatment of conductive or mixed hearing loss, to our knowledge no other case of oticus LP implanted with a BAHD has been reported in the literature. In the reported patient, indeed, the BAHD allowed to achieve satisfactory hearing results, without affecting the ear canal and without the need of a surgical treatment of the occlusion of the ear canal.

In the present article, we report a case with a rare otic localization of LP. In our case also, as previously reported in the scientific literature, topical therapies with antibiotics and corticosteroid eardrops proved to be effective to control the local symptoms.^3^, ^5^, ^6^ Further, this report attests the effectiveness and safety of BAHD implantation for the treatment of the mixed or conductive hearing impairment in cases of oticus LP.

## CONFLICT OF INTEREST

None declared.

## AUTHOR CONTRIBUTIONS

FL: drafted part of the manuscript and reviewed the literature. LB: drafted part of the manuscript and contributed to review the draft. SB: reviewed the draft. ADV: drafted part of the manuscript and helped reviewing the literature. FF: drafted part of the manuscript and contributed to review the draft. All authors: approved this final draft for consideration of publication.
